# Plasma NfL and GFAP as biomarkers of spinal cord degeneration in adrenoleukodystrophy

**DOI:** 10.1002/acn3.51188

**Published:** 2020-10-13

**Authors:** Wouter J. C. van Ballegoij, Stephanie I.W. van de Stadt, Irene C. Huffnagel, Stephan Kemp, Eline A. J. Willemse, Charlotte E. Teunissen, Marc Engelen

**Affiliations:** ^1^ Department of Paediatric Neurology Amsterdam Leukodystrophy Center Emma Children’s Hospital Amsterdam University Medical Centers University of Amsterdam Amsterdam The Netherlands; ^2^ Department of Neurology OLVG Hospital Amsterdam The Netherlands; ^3^ Laboratory Genetic Metabolic Diseases Department of Clinical Chemistry Amsterdam University Medical Centers Amsterdam Gastroenterology & Metabolism University of Amsterdam Amsterdam The Netherlands; ^4^ Neurochemistry lab and Biobank Department of Clinical Chemistry Amsterdam Neuroscience Amsterdam University Medical Centers VU University Amsterdam The Netherlands

## Abstract

**Objective:**

To explore the potential of neurofilament light (NfL) and glial fibrillary acidic protein (GFAP) as biomarkers of spinal cord degeneration in adrenoleukodystrophy, as objective treatment‐outcome parameters are needed.

**Methods:**

Plasma NfL and GFAP levels were measured in 45 male and 47 female ALD patients and compared to a reference cohort of 73 healthy controls. For male patients, cerebrospinal fluid (CSF) samples (n = 33) and 1‐year (n = 39) and 2‐year (n = 18) follow‐up data were also collected. Severity of myelopathy was assessed with clinical parameters: Expanded Disability Status Scale (EDSS), Severity Scoring system for Progressive Myelopathy (SSPROM), and timed up‐and‐go.

**Results:**

NfL and GFAP levels were higher in male (*P* < 0.001, effect size (partial ƞ^2^) NfL = 0.49, GFAP = 0.13) and female (*P* < 0.001, effect size NfL = 0.19, GFAP = 0.23) patients compared to controls; levels were higher in both symptomatic and asymptomatic patients. In male patients, NfL levels were associated with all three clinical parameters of severity of myelopathy (EDSS, SSPROM, and timed up‐and go), while GFAP in male and NfL and GFAP in female patients were not. Changes in clinical parameters during follow‐up did not correlate with (changes in) NfL or GFAP levels. Plasma and CSF NfL were strongly correlated (r = 0.60, *P* < 0.001), but plasma and CSF GFAP were not (r = 0.005, *P* = 0.98).

**Interpretation:**

Our study illustrates the potential of plasma NfL as biomarker of spinal cord degeneration in adrenoleukodystrophy, which was superior to plasma GFAP in our cohort.

## Introduction

Progressive myelopathy affects all men and over 80% of women with X‐linked adrenoleukodystrophy (ALD).^1^, ^2^ ALD is a genetic neurometabolic disorder caused by mutations in the *ABCD1‐*gene leading to a defect in the degradation of very long‐chain fatty acids (VLCFA).^3^, ^4^ VLCFA accumulate in plasma and tissues, including the spinal cord, adrenal cortex, and brain white matter.^5^ The pathology of myelopathy in ALD is characterized by axonal degeneration of the long ascending and descending tracts of the spinal cord.^6^, ^7^ Clinically, it presents in adulthood as a progressive gait disorder due to a spastic paraparesis and sensory ataxia; patients also report sphincter disturbance with urinary and fecal urgency and incontinence.^1^, ^8^ Male patients are affected more severely and at a younger age than female patients.^2^, ^9^ In addition to myelopathy, male patients can develop adrenocortical insufficiency and progressive inflammatory white matter lesions (cerebral ALD).^10^, ^11^ Treatment of the myelopathy of ALD is currently supportive only, but disease modifying therapies are under development. Tools to evaluate the efficacy of these treatments in clinical trials are lacking: molecular biomarkers are not available and clinical parameters of disease severity and progression have important limitations.^1^ Therefore, objective and easily accessible treatment‐outcome parameters for myelopathy in ALD are needed.

Neurofilament light (NfL) and glial fibrillary acidic protein (GFAP) are cytoskeletal proteins of neurons and astrocytes, respectively, that are released in the cerebrospinal fluid (CSF) and blood upon damage of these cells. Until recently, these biomarkers could only be measured in CSF because the assays were not sensitive enough for detection of the much lower concentrations in plasma, but the introduction of the single‐molecule array (SiMoA) assay has enabled reliable quantification in blood samples as well.^12^, ^13^, ^14^, ^15^ NfL and GFAP have been shown to serve as biomarkers of nerve tissue damage in a range of neuroinflammatory and neurodegenerative diseases.^16^, ^17^, ^18^ Among these are diseases with degeneration of the long tracts of the spinal cord, such as hereditary spastic paraplegia (HSP) and amyotrophic lateral sclerosis (ALS).^19^, ^20^ As myelopathy in ALD is characterized by degeneration of the corticospinal tracts and dorsal columns of the spinal cord, we hypothesized that NfL and GFAP could also reflect spinal cord degeneration in ALD. To evaluate this, we measured plasma NfL and GFAP levels in a cohort of male and female ALD patients using SiMoA assay. We compared the levels of patients to healthy controls, determined the association with clinical parameters of disease severity and evaluated changes over 2‐year follow‐up. We hypothesized that NfL would perform better as biomarker than GFAP, because axonal (and not glial) degeneration is the pathological hallmark of myelopathy in ALD.^21^ Also, because myelopathy in women with ALD has a milder disease course, we hypothesized that NfL and GFAP levels would be lower and associations with disease severity weaker in female compared to male patients.

## Methods

### Study design and participants

This study consists of data from two observational cohort studies performed at the Amsterdam University Medical Centers: a prospective observational cohort study in male ALD patients and a cross‐sectional study in female ALD patients. Clinical data of these studies have been previously reported.^1^, ^2^, ^9^

Male ALD patients >16 years of age were prospectively recruited between September 2015 and July 2019. Study visits were embedded in routine clinical care, consisting of a yearly hospital visit with neurological examination and cerebral MR imaging. Participation in the study involved a more extensive neurological examination, additional blood sampling, and optional CSF sampling (lumbar puncture). Female patients (who are not routinely followed for patient care because of the milder disease course without treatable complications like adrenocortical dysfunction) were previously evaluated for a baseline visit between 2008 and 2010; blood samples of this baseline visit were not available. For the current study, all women were invited for a follow‐up visit performed between June 2015 and March 2017. To expand the cohort, women with ALD who were diagnosed at our center after the baseline visit were also recruited. Participation consisted of one hospital visit with venous blood sampling and neurological examination. Patients with active or arrested cerebral ALD (defined as gadolinium‐enhancing or non‐enhancing cerebral white matter lesions, respectively) or a history of a neurodegenerative or neuroinflammatory disease (other than ALD) were excluded from participation. The local Institutional Review Board approved the study protocols (METC 2014_302, METC2015_079, METC 2018_310) and all the participants provided written informed consent.

Reference values for both NfL and GFAP were obtained from an in‐house reference cohort that consisted of healthy volunteers between 18 and 75 years old, who were recruited through public advertising and provided written informed consent. For CSF, only reference values for NfL (and not GFAP) were available.

### Assessment of myelopathy

Male and female patients underwent a detailed neurological history and examination to assess myelopathy, as previously described.^1^, ^9^ They were scored as symptomatic if they had both signs and symptoms of myelopathy, otherwise they were scored as asymptomatic. Clinical outcome measures used to quantify myelopathy were the Expanded Disability Status Scale (EDSS), Severity Scoring system for Progressive Myelopathy (SSPROM), and timed up‐and‐go. The EDSS measures neurological disability ranging from 0 (no disability) to 10 (death).^22^ SSPROM measures the severity of myelopathy ranging from 0 to 100, with lower scores indicating a higher degree of impairment.^23^, ^24^ The timed up‐and‐go is used to assess walking function by recording the time that the patient needs to get up from an armchair, walk 3 meters, turn around, walk back, and sit again.^25^, ^26^

### Sample processing and laboratory methods

Blood was collected in 4‐mL EDTA tubes and processed within 2 hours at the biobank of Amsterdam UMC. Samples were centrifuged for 10 minutes at 2000*g* and plasma was stored at −80°C in 0.5 mL volumes until further use. CSF was collected in 10‐mL polypropylene tubes and centrifuged for 10 minutes at 1800*g*, supernatant was aliquoted in 0.5 mL volumes and stored at −80°C until further use.

Measurements of NfL and GFAP in plasma and CSF were performed in the Neurochemistry laboratory of the Amsterdam UMC location VUmc using the single‐molecule array (SiMoA) technology (Quanterix Corp., MA USA). Analyses were performed using the NF‐light Kit (Quanterix) and GFAP Discovery Kit (Quanterix), run on the SiMoA HD‐1 according to the manufacturer’s protocols (www.quanterix.com/products‐technology/assays). Measurements were performed in duplicate by certified technicians that were blinded to clinical information. The average variation of duplicate measurements was 4.8% for NfL and 3.3% for GFAP.

### Statistical analysis

Baseline characteristics were summarized using descriptive statistics. Normality of the data was assessed by visual inspection of the Q–Q plots and Shapiro–Wilk testing. Data of male and female patients were analyzed separately, as they are known to have a different disease course. To determine whether it was also necessary to subdivide the control group based on gender, we assessed if there were differences in NfL and GFAP levels between male and female controls. Because NfL and GFAP levels are strongly age dependent (both increasing with age), we corrected for differences in age with analysis of covariance (ANCOVA). We assessed if there were differences in NfL and GFAP levels at baseline between 1) patients compared to controls and 2) symptomatic patients, asymptomatic patients, and controls. For comparison of three groups, post hoc testing was performed with Bonferroni correction for multiple comparisons. Although NfL and GFAP levels for the control group were not normally distributed (positively skewed), the standardized residuals were normally distributed, thereby not violating the assumptions of the ANCOVA. For group comparisons in females, controls with age <30 years were excluded to better match the patient group, as there were no female patients represented in this age group. We evaluated the association between the severity of myelopathy and NfL/GFAP levels using multiple linear regression analysis with both age and clinical parameters of severity of myelopathy as independent variables.

We compared baseline CSF NfL values of patients and controls with correction for age (ANCOVA). We determined the correlation between plasma and CSF levels of NfL and GFAP with Spearman’s correlation test (non‐normally distributed data). Because of the relatively low number of available CSF samples, we did not perform comparisons between three groups or correlations with disease severity for CSF data.

For the longitudinal data (male patients only), we calculated mean paired changes in clinical parameters of disease severity and NfL/GFAP levels during follow‐up for both the total patient group and the symptomatic subgroup; statistical significance of these differences was assessed using paired t‐test (normally distributed data) or Wilcoxon signed‐rank test (non‐normally distributed data). We evaluated the association between changes in disease severity and biomarker levels by correlating delta scores of clinical parameters to (delta scores of) NfL/GFAP levels. In addition, to evaluate the variability of NfL and GFAP levels over time, we determined the correlation between biomarker levels of subsequent visit with Spearman’s correlation test.

For all statistical tests a significance level of α = 0.05 (two‐sided) was chosen. Significance levels after Bonferroni corrections were reported separately. IBM SPSS statistics version 26 (IBM Inc.) was used for all statistical analyses.

## Results

In total, 185 samples were analyzed: 105 plasma samples from male patients (45 baseline, 60 follow‐up), 47 plasma samples from female patients (all baseline), and 33 CSF samples from male patients (20 baseline, 13 follow‐up). Seven male patients were excluded because of cerebral ALD and one female patient was excluded because of a history of Parkinson’s disease; otherwise there were no exclusions. Clinical characteristics of both the male and female cohort are described in detail elsewhere and are summarized in Table 1.^1^, ^9^

**Table 1:** Patient baseline characteristics.

	Males (n = 45)	Females (n = 47)
Age, y	44.0 ± 16.7	54.0 ± 12.4
Symptomatic	32 (71%)	25 (53%)
EDSS	3.5 (2.0‐6.0)	3.5 (2.5‐4.0)
SSPROM	87.0 (77.0‐99.0)	88.0 (83.0‐96.0)
Timed up‐and‐go, s	5.1 (3.6‐9.6)	5.3 (4.3‐7.2)

Values are displayed as mean ± SD for normally distributed data and median (interquartile range) for non‐normally distributed data.

EDSS, Expanded Disability Status Scale; SSPROM, Severity Scoring system for Progressive Myelopathy.

The control group consisted of 73 healthy subjects: 36 males (mean age 45.9 ± 11.6 years) and 38 females (mean age 42.3 ± 9.8 years). There was no significant difference in plasma NfL (6.9 versus 5.8 pg/mL, *P* = 0.25) or GFAP (75.2 vs 68.7 pg/mL, *P* = 0.97) levels between male and female controls after correcting for age. Therefore, we decided not to subdivide the control group based on gender.

### Group comparisons

**Figure 1:**
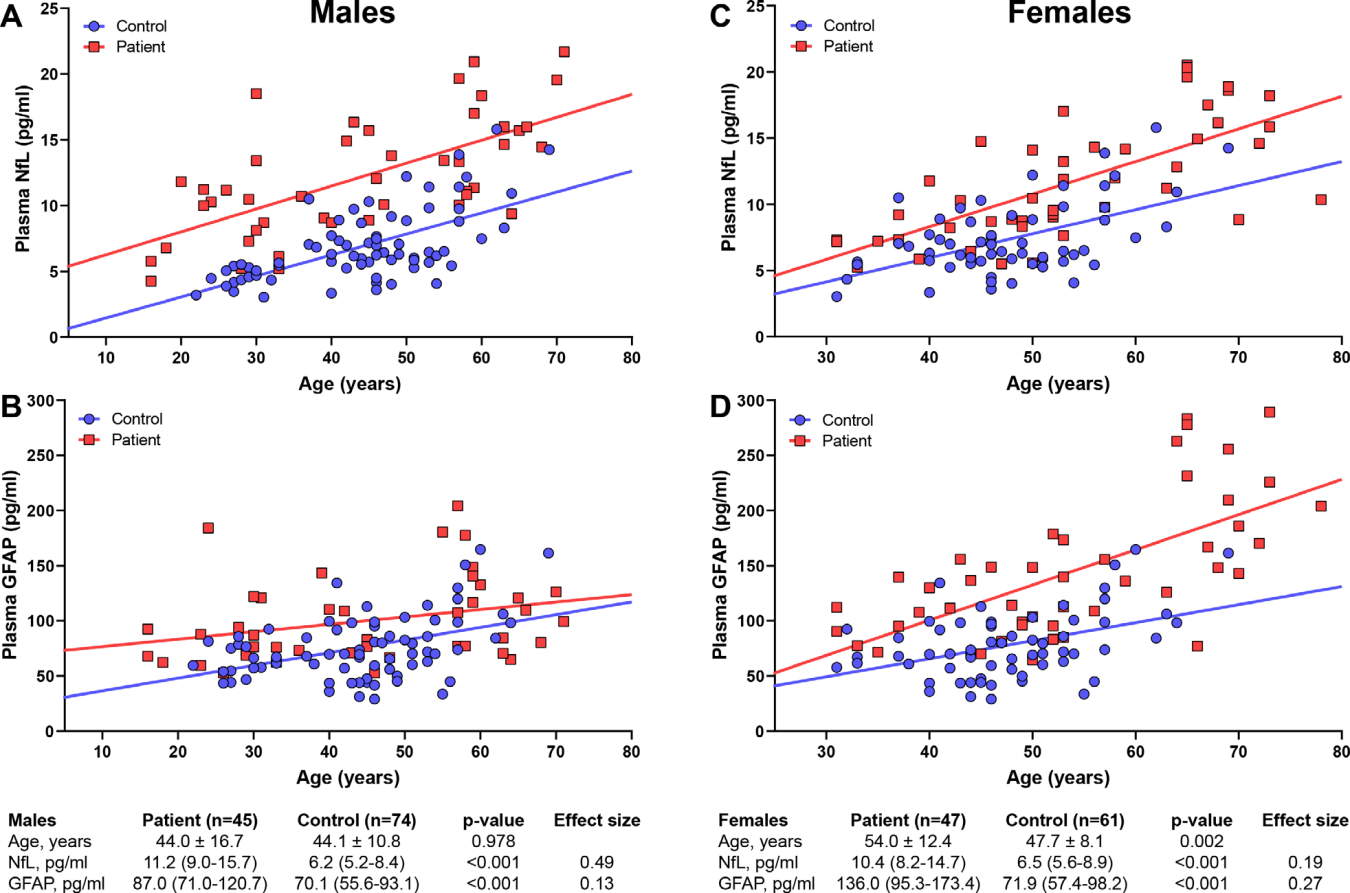
Plasma NfL and GFAP levels in patients versus healthy controls. Graphs show NfL and GFAP levels plotted against age in male (left panel, A and B) and female (right panel, C and D) patients. Values in the tables below the graphs are the median NfL and GFAP levels per group (IQR); p‐values represent the significance level of the difference between groups after correction for age (ANCOVA). Age is displayed as mean ± SD, difference in age was assessed with unpaired t‐test. Effect sizes are the partial eta squared values from the ANCOVA models. GFAP, Glial Fibrillary Acidic Protein; NfL, Neurofilament light.

First, we assessed differences in plasma NfL and GFAP levels between patients and controls (Fig. 1). Mean age of male patients was very similar to the control group (mean difference 0.1 year, *P* = 0.978), while female patients were significantly older compared to controls (mean difference 6.3 years, *P* = 0.002). Age was a significant predictor for NfL levels in both the male (*P* < 0.001, partial η^2^ = 0.42) and female (*P* < 0.001, partial η^2^ = 0.41) model. For GFAP, the association with age was less strong than for NfL but still significant (male *P* < 0.001, partial η^2^ = 0.13; female *P* < 0.001, partial η^2^ = 0.34). After adjustment for age, NfL and GFAP levels were significantly higher in male patients than controls (Fig. 1A and B). Similarly, female patients had significantly higher NfL and GFAP levels than controls (Fig. 1C and D).

Second, we compared plasma NfL and GFAP levels between three groups: controls, asymptomatic patients, and symptomatic patients (Table 2). Asymptomatic patients were significantly younger than symptomatic patients in both the male and female subgroup. For males, after adjustment for age, there was a statistically significant overall difference in NfL (*P* < 0.001, partial η^2^ = 0.50) and GFAP (*P* = 0.001, partial η^2^ = 0.50) levels between groups. Post hoc comparisons showed that levels were significantly higher in both symptomatic patients (NfL *P* < 0.001, GFAP *P* = 0.003) and asymptomatic patients (NfL *P* < 0.001, GFAP *P* = 0.034) compared to controls, but there was no significant difference between asymptomatic and symptomatic patients. For female patients, there was also a statistically significant overall difference in NfL and GFAP levels between groups (Table 2). Similar to male patients, post hoc analysis showed that NfL and GFAP levels were significantly higher in symptomatic (NfL *P* < 0.001, GFAP *P* < 0.001) and asymptomatic (NfL *P* = 0.018, GFAP *P* = 0.001) patients compared to controls, there was no difference between asymptomatic and symptomatic patients.

**Table 2:** Plasma NfL and GFAP levels in controls, asymptomatic patients, and symptomatic patients.

	Control	Asymptomatic	Symptomatic	*P*‐value	Effect size	Post hoc comparisons
Males						
N	74	13	32			
Age, y	44.1 ± 10.8	29.3 ± 13.4	49.9 ± 14.1	<0.001		(C‐A, A‐S)
NfL, pg/mL	6.2 (5.2‐8.4)	8.9 (6.0‐11.2)	13.4 (10.2‐16.3)	<0.001	0.50	(C‐A, C‐S)
GFAP, pg/mL	70.1 (55.6‐93.1)	76.5 (63.7‐93.6)	99.4 (72.7‐121.9)	0.001	0.13	(C‐A, C‐S)
Females						
N	61	22	25			
Age, y	47.7 ± 8.1	45.8 ± 9.7	60.5 ± 10.2	<0.001		(C‐S, A‐S)
NfL, pg/mL	6.5 (5.6‐8.9)	8.9 (7.0‐10.3)	14.2 (9.6‐17.3)	<0.001	0.22	(C‐A, C‐S)
GFAP, pg/mL	71.9 (57.4‐98.2)	109.6 (84.4‐141.7)	148.6 (110.9‐217.6)	<0.001	0.28	(C‐A, C‐S)

Values are displayed as mean ± SD for normally distributed data and median (interquartile range) for non‐normally distributed data. Plasma NfL and GFAP levels are the uncorrected medians (not corrected for age). Kruskal–Wallis test was used to assess between‐group differences in age. ANCOVA was used to assess between‐group differences in NfL and GFAP levels, p‐values represent the significance level after correction for age. Post hoc comparisons indicate which groups significantly differ from each other after Bonferroni correction for multiple comparisons. Effect sizes are the partial eta squared values from the ANCOVA models.

A‐S, asymptomatic versus symptomatic; C‐A, control versus asymptomatic; C‐S, control versus symptomatic; GFAP, Glial Fibrillary Acidic Protein; NfL, neurofilament light.

### Association of disease severity with NfL and GFAP levels

**Figure 2:**
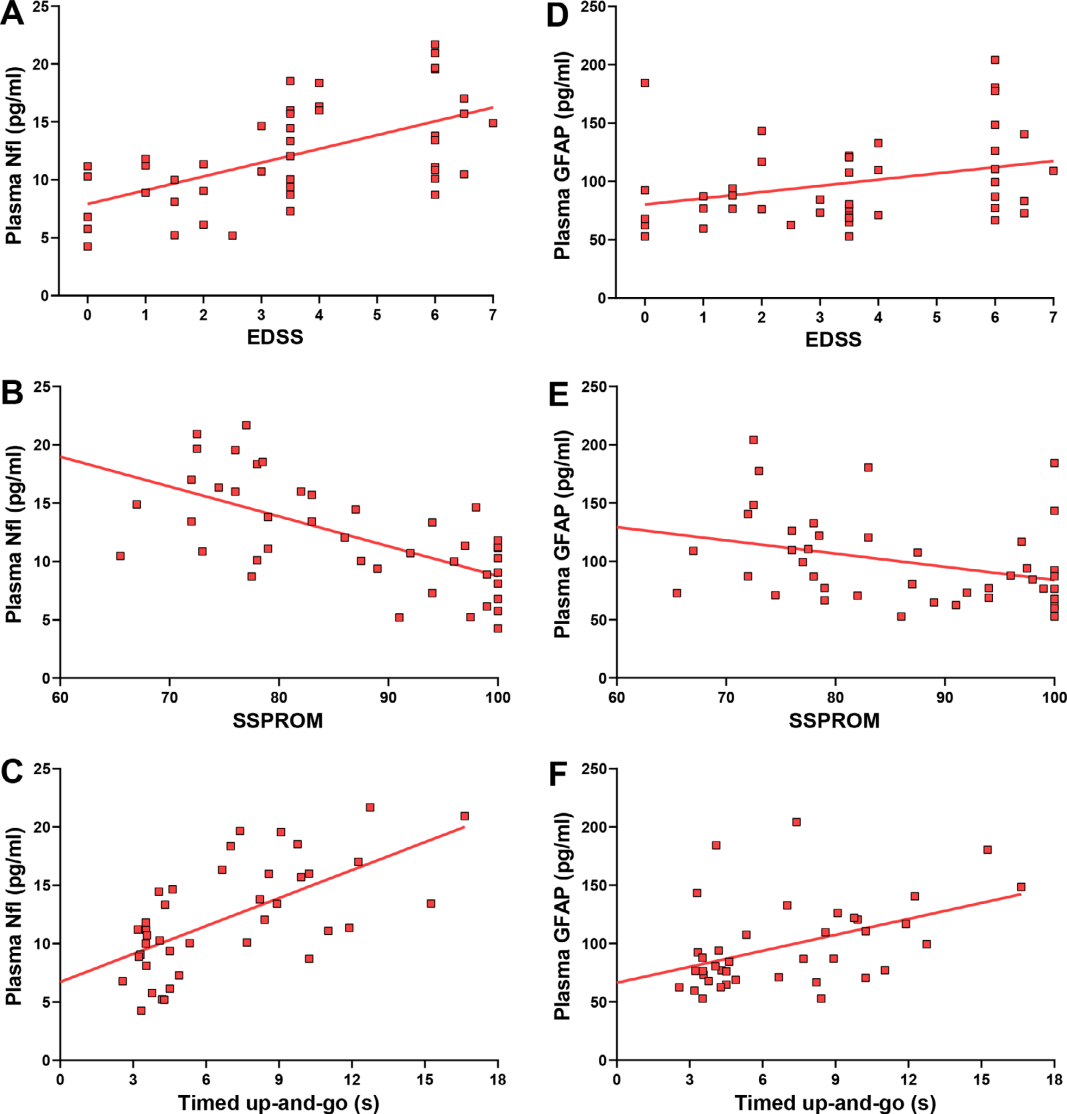
Associations between clinical parameters of severity of myelopathy and plasma NfL levels in male patients. Lines represent simple linear regression lines. EDSS, Expanded Disability Status Scale; GFAP, Glial Fibrillary Acidic Protein; NfL, Neurofilament light; SSPROM, Severity Scoring system for Progressive Myelopathy.

We evaluated the association between severity of myelopathy and biomarker levels by performing multiple linear regression analysis with age and 1) EDSS, 2) SSPROM, and 3) timed up‐and‐go as predictors. As expected, age and clinical parameters of severity of myelopathy were correlated (correlation coefficient between 0.48 and 0.64), but the correlation was below the regularly used cutoff value for collinearity (correlation coefficient >0.8).^27^ In male patients (Fig. 2), all three models significantly predicted plasma NfL levels. For the first model, both age (B = 0.13, *P* = 0.001) and EDSS (B = 0.63, *P* = 0.027) were significant predictors. Similarly, for the second model both age (B = 0.117, *P* = 0.001) and SSPROM (B = −0.17, *P* = 0.001) and for the third model both age (B = 0.122, *P* = 0.002) and timed up‐and‐go (B = 0.47, *P* = 0.009) significantly predicted NfL levels. On the contrary, neither age nor any of the clinical parameters were significant predictors of plasma GFAP levels.

For the female subgroup, age was a significant predictor for NfL and GFAP levels in all three models, but none of the clinical parameters were.

### CSF data

Details of CSF data are presented in Table 3. CSF NfL levels were significantly higher in patients than controls. There was a strong correlation between CSF and plasma NfL levels (Spearman’s rho = 0.60, *P* < 0.001). Plasma and CSF levels of GFAP were not correlated (Spearman’s rho = 0.005, *P* = 0.98). Unfortunately, CSF GFAP data of healthy controls were not available.

**Table 3:** Baseline CSF NfL and GFAP levels in patients and controls.

	Patient (n = 20)	Control (n = 49)	*P*‐value
Age, y	47.5 (30.0‐57.0)	54.0 (46.5‐60.5)	0.034
Symptomatic	30 (70%)		
EDSS	5.0 (2.0‐6.0)		
SSPROM	82.5 (77.6‐98.5)		
Timed up‐and‐go, s	8.0 (4.3‐10.6)		
NfL, pg/ml	752.5 (665.3‐1042.1)	642.4 (585.9‐743.8)	0.001
GFAP, pg/ml	5156.9 ± 2097.3		

Values are displayed as mean ± SD for normally distributed data and median (interquartile range) for non‐normally distributed data. NfL and GFAP levels are the uncorrected medians (not corrected for age). Kruskal–Wallis test was used to assess between‐group differences in age. ANCOVA was used to assess between‐group differences in NfL levels; the p‐values represent the significance level after correction for age. GFAP data for healthy controls were not available.

GFAP, Glial Fibrillary Acidic Protein; CSF, cerebrospinal fluid; EDSS, Expanded Disability Status Scale; NfL, neurofilament light; SSPROM, Severity Scoring system for Progressive Myelopathy.

### Longitudinal data

Follow‐up samples were available for 39 of 45 (87%) patients for year 1 and 18 of 45 (40%) patients for year 2. There was a small increase in the EDSS during follow‐up (mean paired change 0.41, *P* = 0.041), but SSPROM and timed up‐and‐go did not change (Supplementary Table S1). NfL and GFAP levels did not change significantly during follow‐up. There were no correlations between changes on clinical parameters and (changes on) NfL/GFAP levels (correlation coefficients <0.3).

**Figure 3:**
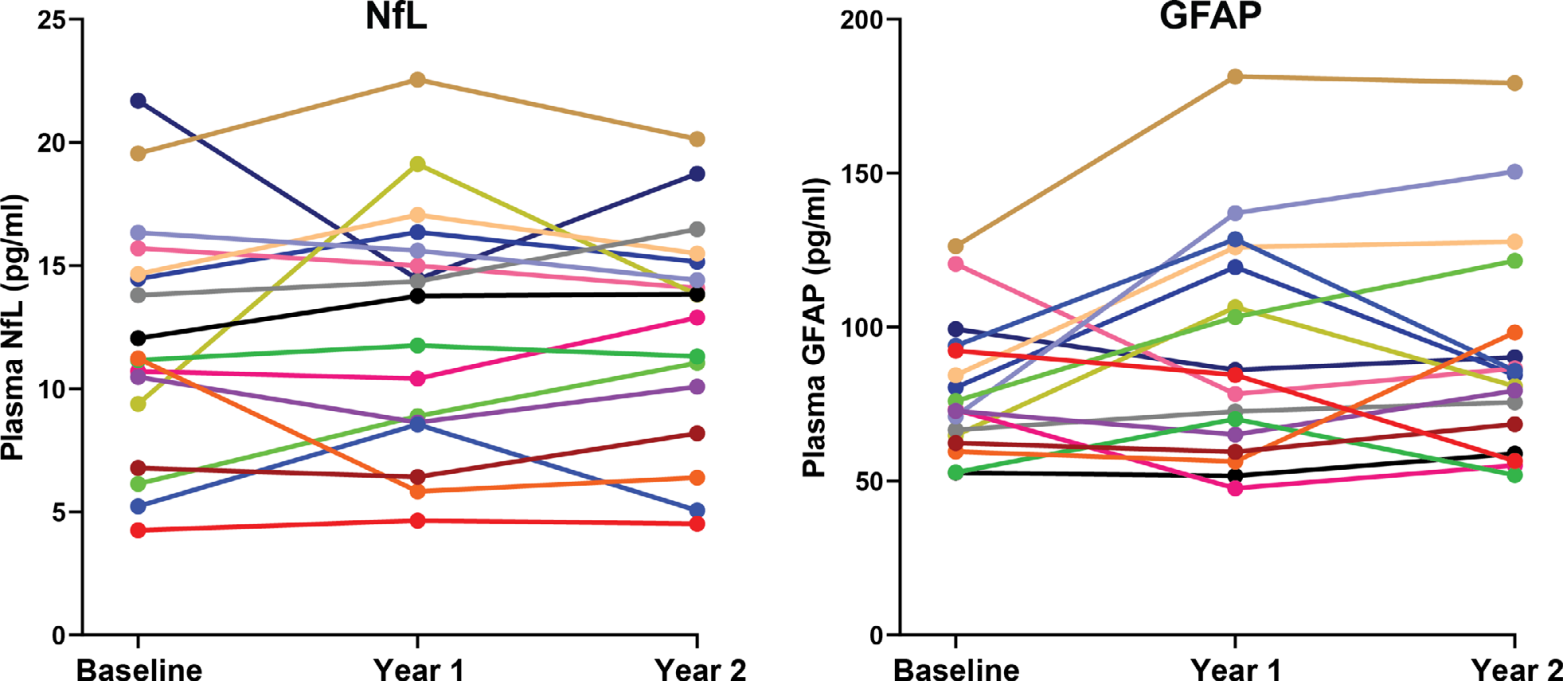
NfL (A) and GFAP (B) levels during follow‐up for the 18 male patients that completed all three visits. GFAP, Glial Fibrillary Acidic Protein; NfL, Neurofilament light.

Biomarker levels for patients that completed all three visits are represented in Figure 3. To evaluate the variability of NfL and GFAP levels over time, we determined the correlation between biomarker levels at subsequent visits. For NfL, levels at baseline and year 1 correlated strongly (Spearman’s rho = 0.79, *P* < 0.001), as did levels at year 1 and year 2 (Spearman’s rho = 0.88, *P* < 0.001). For GFAP, correlations between baseline and year 1 (Spearman’s rho = 0.75, *P* < 0.001) and between year 1 and year 2 (Spearman’s rho = 0.69, *P* = 0.002) were also strong, albeit less strong than for NfL.

## Discussion

As disease modifying therapies for myelopathy in ALD are under development, there is a need for reliable, observer‐independent and easily accessible treatment‐outcome parameters. In this explorative study we demonstrate that NfL could serve as a biomarker of spinal cord degeneration in ALD, while GFAP seems less valuable. Both plasma NfL and GFAP levels were significantly elevated in patients compared to healthy controls, but only NfL levels in males were associated with clinical parameters of disease severity. We found no correlations between (changes in) biomarker levels and parameters of disease progression.

We hypothesized that NfL would be a better biomarker for spinal cord degeneration than GFAP, since axonal rather than glial degeneration is the pathological hallmark of myelopathy in ALD.^7^ Indeed, data from our study to support NfL as biomarker are much more robust than for GFAP. First, one would expect a biomarker of spinal cord degeneration to be higher in male than female ALD patients, as male patients are more severely affected with an earlier disease onset and faster progression. Group differences in NfL levels were indeed larger for male than female patients (Fig. 1, Table 2). For GFAP, we found exactly the opposite, with larger group differences in female than male patients (Table 2). This can be partially explained by the difference in age, as female patients in our study were on average 10 years older than male patients. But even after correction for the difference in age, GFAP levels in female patients were higher, for which we do not have a pathophysiological explanation. Second, NfL levels were associated with each of the three clinical measures of disease severity (with more severely affected patients having higher NfL levels) in males, supporting its role as biomarker of spinal cord degeneration, while these associations were not present for GFAP. Finally, the correlation between CSF and plasma levels of NfL was strong (correlation coefficient 0.60) and comparable to other studies,^12^, ^28^, ^29^ while we found no correlation between CSF and plasma levels of GFAP (correlation coefficient 0.005).

There is an important difference between molecular biomarkers such as NfL and GFAP and other (surrogate) outcomes used for myelopathy in ALD. Most outcomes – for example clinical parameters (EDSS, SSPROM, timed walking activities) or imaging biomarkers (spinal cord atrophy, diffusion tensor imaging) – represent disability or accumulated spinal cord damage resulting from years of spinal cord degeneration.^1^, ^30^, ^31^, ^32^ NfL and GFAP – with an estimated half‐life of a number of days and months respectively – reflect current or recent neurodegeneration and are therefore the markers of ongoing or recent disease activity.^33^, ^34^ This makes the relationship between NfL and severity of myelopathy not straightforward. For example, a young patient could have severe spinal cord degeneration with elevated NfL levels, while not (yet) having any disability. This theory is supported by our finding that NfL was elevated to a similar degree in asymptomatic patients as in symptomatic patients, suggesting that spinal cord degeneration in the asymptomatic group is already ongoing but has not yet resulted in enough damage to cause symptoms or disability. It is likely that a certain threshold of neurodegeneration has to be reached before symptoms of myelopathy appear. If this hypothesis is true, NfL could be used to monitor disease activity in presymptomatic patients, for whom markers of disability do not apply.

The relationship between NfL and myelopathy in ALD is further complicated by the confounding effect of age. Normal aging is associated with neurodegenerative processes that cause NfL levels to increase with age.^16^ Myelopathy in ALD is also age dependent: symptoms start on average in early adulthood and slowly progress, with most patients losing unassisted ambulation by the 6th decade.^8^ Consequently, the associations between disease severity and NfL levels we found (Fig. 2) are partly explained by aging. However, even after taking this effect of age into account (by multiple regression analysis with both age and severity of myelopathy as predictors), severity of myelopathy was still a significant predictor of NfL levels.

Group differences in NfL levels and correlations with disease severity support the use of NfL as biomarker for ALD. However, in order to prove that NfL is a surrogate marker for spinal cord degeneration, it is necessary to demonstrate that elevated NfL levels lead to (progression of) myelopathy, while low NfL levels do not. In our cohort, we did not find a correlation between NfL levels and clinical disease. Disease progression was probably not substantial enough (with only minimal change on the EDSS and not on the other clinical parameters, Supplementary Table S1) to be able to demonstrate such a correlation. This is likely due to the inherent slow progression of myelopathy in ALD, low sensitivity of the clinical parameters in detecting disease progression,^1^ and a relatively low number of patients with complete follow‐up. Longer follow‐up, which is ongoing in this cohort, might resolve this issue.

NfL has several potential advantages as biomarker in ALD. Being a marker of disease activity, NfL could show an effect of a disease modifying treatment on a short term, while currently available clinical endpoints require very long follow‐up. Although phase III trials usually require clinical endpoints, NfL could be particularly useful for phase II trials to identify drugs that seem promising enough to continue to phase III trials – similar to its application in multiple sclerosis (MS).^16^, ^35^ In addition, it is easily accessible (it can be collected during routine blood sampling), inexpensive, observer independent, and very reproducible provided that samples are processed in the same laboratory.^12^, ^13^ Main disadvantage is that it is a general biomarker for axonal degeneration, which is not specific for ALD. Other neurological disorders – for example recent stroke, head trauma, Alzheimer or Parkinson’s disease – also lead to elevated NfL levels and are an important source of bias.^18^, ^36^ Therefore, if NfL is to be used as treatment‐outcome parameter, it is important to screen for these conditions and exclude patients if necessary.

In conclusion, our study illustrates the potential of NfL as a biomarker of spinal cord degeneration in male ALD patients, while plasma GFAP seems less valuable. NfL could serve as a surrogate outcome in phase II trials or as secondary outcome in phase III trials. A longitudinal study demonstrating that elevated NfL levels lead to progression of myelopathy is needed to confirm our findings and is currently ongoing in this cohort.

## Conflict of Interest

The authors declare the following potential conflict of interest: IH has received unrestricted research grants from Vertex. ME has received unrestricted research grants from Vertex, Swanbio Therapeutics, Bluebird Bio, and Minoryx Therapeutics. SK received consulting fees from SwanBio Therapeutics and unrestricted research grants from Swanbio Therapeutics and Bluebird Bio. No other potential conflict of interest apply.

## Supporting information


**Supplementary Table S1.** Changes in NfL, GFAP, and clinical parameters of severity of myelopathy during follow‐up. Values are displayed as mean ± SD for normally distributed data and median (interquartile range) for non‐normally distributed data. Changes during follow‐up were assessed with paired t‐test for normally distributed data and Wilcoxon signed‐rank test for non‐normally distributed data. EDSS, Expanded Disability Status Scale; GFAP, Glial Fibrillary Acidic Protein; NfL, neurofilament light; SSPROM, Severity Scoring system for Progressive Myelopathy (SSPROM).
