# Supplementary material for: Plasma NfL and GFAP as biomarkers of spinal cord degeneration in adrenoleukodystrophy
Source: Ann Clin Transl Neurol. 2020 Oct 13;7(11):2127–36. doi: 10.1002/acn3.51188 (PMC7664277; doi:10.1002/acn3.51188)
Supplement: Supplementary file 1 — Supplementary Table S1. Changes in NfL, GFAP, and clinical parameters of severity of myelopathy during follow‐up. Values are displayed as mean ± SD for normally distributed data and median (interquartile range) for non‐normally distributed data. Changes during follow‐up were assessed with paired t‐test for normally distributed data and Wilcoxon signed‐rank test for non‐normally distributed data. EDSS, Expanded Disability Status Scale; GFAP, Glial Fibrillary Acidic Protein; NfL, neurofilament light; SSPROM, Severity Scoring system for Progressive Myelopathy (SSPROM). [file ACN3-7-2127-s001.docx]

|  | **Baseline** | **Year 2** | **Mean paired**  **change (95%CI)** | **p-value** |
| --- | --- | --- | --- | --- |
| **All patients** |  |  |  |  |
| EDSS | 3.5 (1.3-5.0) | 3.5 (1.0-6.0) | 0.41 (0.01-0.82) | **0.041** |
| SSPROM | 89.0 (78.0-99.5) | 87.0 (77.0-100.0) | -0.62 (-2.31-1.08) | 0.754 |
| Timed up-and-go, s | 4.5 (3.5-8.4) | 4.3 (3.4-9.0) | 0.24 (-0.33-0.81) | 0.569 |
| Nfl, pg/ml | 12.0 ± 4.9 | 12.4 ± 4.5 | 0.47 (-0.79-1.74) | 0.441 |
| GFAP, pg/ml | 73.2 (63.6-93.2) | 84.5 (63.7-109.9) | 11.88 (-4.14-27.92) | 0.177 |
|  |  |  |  |  |
| **Symptomatic only** |  |  |  |  |
| EDSS | 3.75 (3.5-6.0) | 6.0 (3.5-6.0) | 0.55 (-0.13-1.23) | 0.109 |
| SSPROM | 80.9 ± 8.0 | 79.8 ± 10.0 | -1.15 (-4.11-1.82) | 0.404 |
| Timed up-and-go, s | 7.5 ± 3.0 | 8.1 ± 3.5 | 0.64 (-0.30-1.58) | 0.152 |
| Nfl, pg/ml | 14.4 ± 4.0 | 15.0 ± 2.9 | 0.55 (-1.10-2.20) | 0.472 |
| GFAP, pg/ml | 73.0 (66.2-104.7) | 82.7 (75.5-90.3) | 11.31 (-12.35-34.96) | 0.445 |

**Supplementary Table 1.** Changes in Nfl, GFAP and clinical parameters of severity of myelopathy during follow-up.

Values are displayed as mean ± SD for normally distributed data and median (interquartile range) for non-normally distributed data. Changes during follow-up were assessed with paired t-test for normally distributed data and Wilcoxon signed rank test for non-normally distributed data.

EDSS, Expanded Disability Status Scale; GFAP, Glial Fibrillary Acidic Protein; Nfl, neurofilament light, SSPROM, Severity Scoring system for Progressive Myelopathy (SSPROM).
